# Exfoliation Syndrome in Baja Verapaz Guatemala: A Cross-Sectional Study and Review of the Literature

**DOI:** 10.3390/jcm11071795

**Published:** 2022-03-24

**Authors:** Chase Paulson, Samuel C. Thomas, Orlando Gonzalez, Samuel Taylor, Cole Swiston, Jennifer S. Herrick, Lori McCoy, Karen Curtin, Craig J. Chaya, Brian C. Stagg, Barbara M. Wirostko

**Affiliations:** 1 School of Medicine, University of Utah, Salt Lake City, UT 84132, USA; chase.paulson@hsc.utah.edu (C.P.); sam.taylor@hsc.utah.edu (S.T.); jennifer.herrick@hsc.utah.edu (J.S.H.); karen.curtin@hsc.utah.edu (K.C.); 2 Department of Medicine and Population Health, School of Medicine, Stanford University, Palo Alto, CA 94304, USA; sthomas4@stanford.edu; 3 Lions Club Eye Hospital, Salamá 150001, Guatemala; orlandgpicado@gmail.com; 4 Moran Eye Center, University of Utah, Salt Lake City, UT 84132, USA; cole.swiston@hsc.utah.edu (C.S.); lori.mccoy@hsc.utah.edu (L.M.); craig.chaya@hsc.utah.edu (C.J.C.); brian.stagg@hsc.utah.edu (B.C.S.)

**Keywords:** pseudoexfoliation syndrome, glaucoma, Guatemala, underserved, minority, UV exposure, Salamá

## Abstract

There are little epidemiologic data on exfoliation syndrome (XFS) or exfoliation glaucoma (XFG) in Guatemala, especially in the underserved Baja Verapaz region. This observational study assessing XFS/XFG and demographic factors of this region aims to better understand unique exogenous and endogenous risk factors associated with XFS/XFG in Guatemala. During Moran Eye Center’s global outreach medical eye camps from 2016–2017, 181 patients age 15 years and older presented for complete eye exams. These individuals were screened for eye disease and evaluated for possible surgical interventions that could occur during the camps to improve eyesight. During the dilated exams, XFS was noted as missing or present. Of those 181, 10 had insufficient data and 18 lacked a definitive diagnosis of XFS or XFG, resulting in 153 evaluable patients; 46 XFS and 9 XFG were identified. Age, gender, hometown, ancestry (languages spoken by parents and grandparents), past medical history, family medical history, and occupational data (only 2017 trip) were obtained for each patient. The most common occupations of these individuals were farming and housekeeping. Higher rates of XFS/XFG were noted in individuals of rural compared to urban settings and Mayan speaking people compared with Spanish speakers. Based on this subset of patients, with various ocular pathologies being evaluated during medical eye outreach camps, the prevalence of XFS/XFG appeared to be 36%, a high prevalence compared to other world populations. Location and higher altitude, along with a farming occupation, may contribute to XFS development and subsequent progression to XFG. To our knowledge, this is the largest study looking at the epidemiology of XFS/XFG in the Baja Verapaz region of Guatemala for those over the age of 15 years seeking eye exams and interventions.

## 1. Introduction

Guatemala is one of the most underserved countries in Central America and close to half of Guatemalans live in rural areas [1]. In addition to many of Guatemala’s citizens inadequate access to housing, clean water, food, and education, they also lack access to health care and especially eye care [2]. There are estimates that of 80,000 Guatemalans are blind due to cataracts and thousands more are functionally blind from lack of access to eyeglasses [3,4]. In the mountainous region of Baja Verapaz, located several hours north of Guatemala City is the city of Salamá, people of diverse languages and communities call this home, including the majority of Mayan Guatemalans that live in the highlands [5]. The vast majority of people residing in Salamá and nearby regions are medically underserved as there is only one ophthalmologist for roughly 800,000 inhabitants. Obtaining population-based data of eye diseases that affect the underserved population in Guatemala will help with the development of interventions for preventable blindness in this population, thereby having a profound impact on quality of life and socioeconomic benefits [6,7].

**Figure 1:**
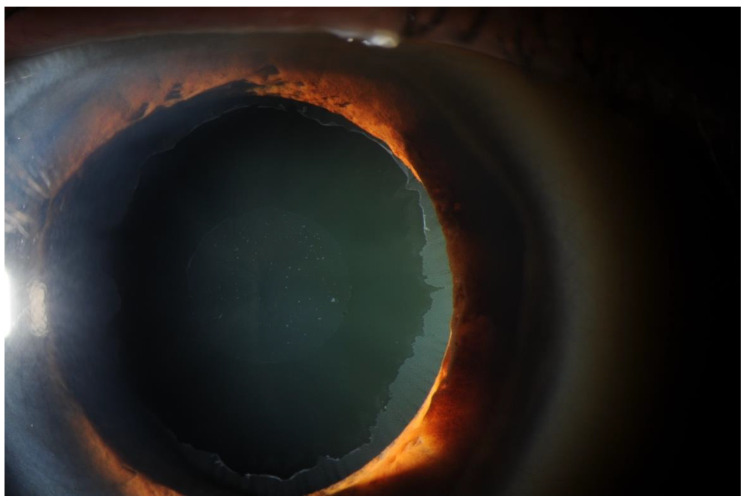
Image of the anterior segment of an eye with XFS.

Exfoliation syndrome (XFS), first discovered in 1917 in a Finnish population, is a complex, inherited systemic disorder characterized by abnormal accumulation of extracellular matrix material (ECM) in the eye, heart, brain, lungs, and skin [8,9,10,11,12,13,14,15]. Deposition of fibrillar ECM debris, or exfoliation material (XFM), within anterior segment structures of the eye is the manifestation of clinically diagnosed XFS (see Figure 1), which is the most common recognizable cause of open-angle glaucoma worldwide [16]. Patients with XFS are at high risk of developing exfoliation glaucoma (XFG), a particularly aggressive form of glaucoma, as well as more advanced and rapid cataract formation [17]. It is important to diagnose XFS given that cataract surgery in XFS can carry a higher risk of intraoperative and postoperative complications [18,19].

Although originally believed to be an inherited disease found primarily in Scandinavian descendants, the XFS phenotype has been reported in multiple populations and appears to be highly associated with genetic variants in the lysyl oxidase like-1 (LOXL1) locus, a key enzyme in ECM deposition and repair [20,21,22,23]. Several additional hypotheses exist as to why this XFM collects and deposits in certain patients based on epigenetic underpinnings, possibly due to DNA methylation [24], UV exposure [25,26,27], and the latitude of a population [27]. The Reykjavik Eye Study [28] found no relation between time outdoors and risk of XFS but, recently, cumulative solar exposure and outdoor occupation have been linked to XFS development in USA and Israeli patients [26,29].

Based on the literature, XFS is rarely observed below age 50, and the prevalence increases significantly with age [30]. In a study of subjects over 60 years across various ethnicities, the prevalence rates ranged between 0% in Greenland Eskimos to 21% in Icelanders [31]. Other XFS prevalence rates include 0.4% in China [32], 13% in Spain [33], 7.8–12% for Gurung and 0.3% for Tamang peoples in Nepal [34], and 2.4% in women over the age of 65 in our Utah population [35]. One study found an XFS prevalence of 15% in western Guatemala and another found 24% in Baja Verapaz (22% had XFS and cataracts), but there are little epidemiologic data on XFS and XFG prevalence or incidence in Guatemala, especially in the Baja Verapaz region [36,37,38]. This observational study assessing XFS, XFG, and possible demographic factors of this region aims to better understand unique exogenous and endogenous risk factors that may be associated with XFS in Guatemalans.

## 2. Materials and Methods

During Moran Eye Center’s global outreach trips from 2016–2017, the study team, working in conjunction with the Salama Lions Eye Hospital staff, traveled to the Baja Verapaz region to offer eye care and assess the prevalence of XFS/XFG in an underserved population. In Salama and several surrounding communities, outreach eye clinics were established to offer complete eye exams, refractions, and screening of patients for eye diseases that would benefit from surgery. People from all over the Baja Verapaz and the surrounding region traveled to these clinics, some more than 50 miles, to receive comprehensive dilated eye exams. All community members who presented to outreach clinics were seen and evaluated. In total, 181 patients were recruited for the study and consented. Access to care was not denied to any community member who came to the clinics. Study participants ranged in age from 15 to 94 years of age. Participants who could benefit from surgery received surgery, i.e., cataract, glaucoma, or pterygium, during those trips. Those with XFS/XFG were identified on dilated slit lamp exam. Glasses were made for those patients with refractive errors and all patients were referred back to the local ophthalmologist in Salamá for continued care.

At these on sight medical camps in the field, Moran surgeons and teams provide free eye exams as well as surgical intervention that can restore sight to hundreds of patients in a week, while also helping local trainees gain experience. During these time periods, the Moran outreach team was in Guatemala from 2014–2017. The Moran Outreach, a team consisting of ophthalmologists, nurses, medical and surgical technicians, and researchers, is an investment in training more ophthalmologists around the world.

Under informed consent, which was translated into either Spanish or Mayan, eye exams were performed, questionnaires were completed, and blood samples for future genetics work from XFS patients and their family members were collected. Age, gender, hometown, ancestry (languages spoken by parents and grandparents), past medical history (PMH), family medical history (FH), and occupational (only 2017 trip) data were obtained for each patient. Location/hometown was further divided into rural (less than 2000 inhabitants) and urban (>2000 inhabitants). Patients were allowed multiple responses for language, PMH and FH. This research was conducted under Utah IRB (00081512). All data were de-identified, HIPAA compliant, and adhered to the tenets of the Declaration of Helsinki.

In 2016, anterior lens capsules were collected on those patients undergoing cataract surgery. These capsules were processed under a collaborative agreement with Singapore Research Center and Duke University looking to measure mRNA for LOXL1.

Demographic variables were examined using descriptive statistics. To determine if there were any significant differences between patients with and without an XFS/XFG diagnosis, a chi-squared test was used for all discrete variables and the Kruskal–Wallis test was used for age. To account for multiple testing, the Bonferroni correction was applied. Demographic variables that were significantly associated with an XFS/XFG diagnosis after adjustment for multiple testing were considered for inclusion in multivariable modeling. The resulting multivariable logistic regression model was adjusted for age and no family medical history. *p*-values ≤ 0.05 were considered statistically significant. All analysis were performed using SAS version 9.4 (SAS Institute, Cary, NC, USA).

## 3. Results

**Figure 2:**
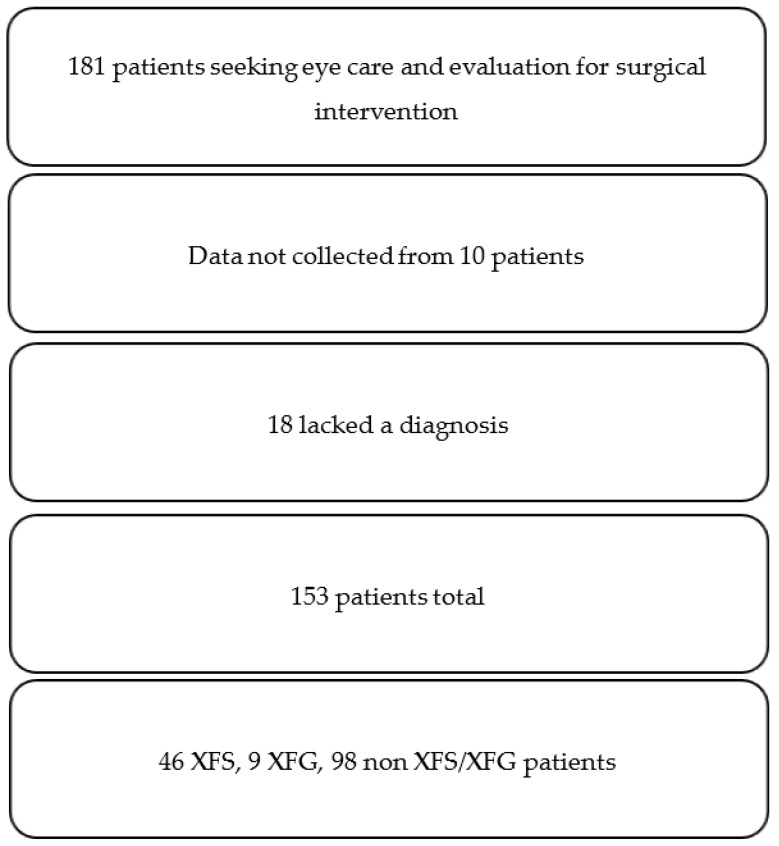
Study Flowchart.

Out of 181 total patients evaluated by the Moran team who provided consent to join the study, 10 patients had insufficient data to be included and 18 lacked a clear diagnosis of either XFS or XFG, resulting in 153 viable patients (Figure 2). The overall prevalence of XFS/XFG in our studied population appeared to be 36%. Of these 153 patients, 66 were male (45%), 81 were female (55%), and 6 did not indicate gender. The XFS/XFG rate in males was 52%, with a rate of 48% in females, and XFS/XFG patients were 48 years of age or older. Demographic details are included in Table 1.

The average age of all patients being examined was 64 years with a range of 15 to 94 years. Those 55 patients with XFS/XFG were, on average, 72 years of age. The median age in years for XFS/XFG patients is significantly older than non XFS/XFG patients at 74 and 63, respectively, with a *p*-value = 0.0008. A significantly higher proportion of patients with an XFS/XFG diagnosis (32 (58%)) compared to no XFS/SFG diagnosis (30 (31%)) had no reported family medical history, *p*-value 0.02.

Lack of any reported family history significantly increased the odds of an XFS/XFG diagnosis in both the unadjusted (OR 3.15, 95% CI 1.59, 6.27, *p*-value 0.001) and adjusted analysis (OR 2.34, 95% CI 1.13, 4.83, *p*-value 0.02). Details of univariate and multivariate analysis can be found in Table 2.

The most common occupations were farming and housekeeping. Higher rates of XFS/XFG were noted in individuals of rural (76%) compared to urban settings (24%). Self-proclaimed ethnicity in Mayan and Spanish people were 40% and 51%, respectively. Blood analysis is underway. Capsular analysis for LOXL1 mRNA was negative.

XFS/XFG rates in parental language was 64% for Spanish and 44% languages other than Spanish. Rates for grandparent language were similar at 56% Spanish and 45% Mayan. Rates of XFS/XFG patients had three salient conditions in their PMH and FH: cardiovascular (CV) disease, hypertension (HTN), and diabetes (DM). PMH rates were 6% for DM and 19% HTN, FH was 7% DM, and 11% CV disease and HTN in the XFS/XFG cohort. Further details on occupation, PMH, and FH can be found in Table 1.

## 4. Discussion

Although XFS was initially identified primarily in individuals of Scandinavian descent, it has since been found in diverse populations worldwide [8]. Reported prevalence of XFS varies significantly across populations, with 0% in Eskimos [31], 0.8% in Kathmandu (Nepal) [39], 1.8% USA [40], 6.45% in Pakistan [41], 7.4% in India [42], 9.6% in Iran [43], 5–25% in Scandinavian countries [31], and 0.6% [44] and 38% in Navajo Indians [45]. Minimal XFS data exist in Latin American populations besides a single case in Brazil [46], a prevalence of 14.5% in Argentina [47], 15% in a western Guatemalan cataract population [36], and 24% in a Mayan cohort (22% had XFS and cataracts) [38]. Our specific Guatemalan population presenting to outreach eye camps to assess their vision and for possible surgical intervention appears to have a high prevalence of XFS/XFG of 36%.

In this population of patients presenting to outreach eye clinics, XFS was found in higher proportions of patients with advanced age, males, rural locations, outdoor occupation, non-Spanish languages spoken by parents and grandparents, and a PMH or FH of HTN, DM, and CV disease. CV disease included arrhythmia, heart attack, heart disorder, dyslipidemia, and stroke, which are chronic conditions generally associated with underlying atherosclerosis [48]. Recently, CV disease was determined to be an associated risk factor in influencing early glaucoma development in XFS patients, perhaps due to vascular dysfunction [49,50].

While age was statistically significantly associated with XFS/XFG, the increase in the odds of an XFS/XFG diagnosis was a very modest increase of 6%. The association of XFS/XFG with lack of family history of CV disease, asthma, and diabetes is very strong. Two studies suggest that XFS might be associated with longer survival [13,14]. A possibility for this is patients with XFS/XFG may have the XFS favorably altering the relation between other systemic diseases and survival. This possible interaction is not currently well-understood and research to clarify this relationship should be conducted.

In this XFS/XFG cohort, approximately equal numbers of XFS were seen in those who reported Mayan versus Spanish descent (40% and 51%, respectively). This could be due to a stronger environmental rather than genetic variability in this region. Further, the difference in XFS rates between sexes of 52% males and 48% females is consistent with previously published literature [51,52], and, in this scenario, could be attributed to cultural norms of men having outdoor occupations like farming, thus increasing their UV exposure.

Various environmental and epigenetic factors have been hypothesized to increase the risk of XFS, such as ocular exposure to UV light and increasing latitude and altitude leading to epigenetic modifications [26,27]. UV exposure is an interesting risk factor to consider in this population given their ample UV exposure due to the predominant occupations of rural regions like Baja Verapaz. It has been hypothesized that UV could likely be related to XFS as a cause of tissue insult (oxidative stress), an epigenetic risk factor, triggering molecular pathways that in turn perform and result in abnormal extracellular matrix accumulation [53].

The environmental risk factor of increased sun exposure could be contributory to elevated XFS rates in rural communities and those with outdoor occupations. Similarly, XFS was found in 110 of the 480 fisherman or agriculturalists and none of the 60 urban residents in the Northern Adriatic Sea [54]. A study in southern India found that subjects who worked outdoors had a significantly higher odds ratio of XFS [25,55]. It is important to consider the low XFS prevalence of Innuits of Greenland and Peruvian residents of Lake Titicaca (4000 m, mostly sunny, low humidity) [52,56]. Pasquale et al. speculates these groups have relatively thick irides that may ameliorate uveal tract damage caused by the expected high degree of reflected UVR [26]. In our Guatemalan cohort, higher rates of XFS were noted in rural patients, with 76% compared to urban 24%. We hypothesize that increased sun exposure associated with outdoor farming occupations that dominate rural communities in Guatemala may contribute to this increased development of XFS.

Latitude and altitude have also been reported as possible contributors to XFS development. In 1973, Forsius found no XFS in Eskimos but did find XFS in 20% of Lapps living at the same latitude [57,58]. In 2011, Stein et al. determined that living at more northern latitudes within the United States and solar exposure may be an environmental risk factor for XFS [27]. Kang et al., in 2012, similarly found that northern latitudes in the US may contribute to XFS, but Scandinavian heritage was not significantly associated with the disease [59]. Moreover, XFS was found to be more prevalent in people living at high altitudes in two series [60,61], but not in a third [31]. Faulkner found a 38% XFS prevalence in Navajo Indians over age 60 residing at 1500 m, 36° N in Arizona [45], but this has been debated based on a recent pending study at our institution, which found rates of only 0.6% [44]. Stein found, in the US Midwest, that increasing elevation with increased sunny days had a decreased hazard ratio for XFS [27]. Another consideration is Barger et al., who reported a 15% XFS prevalence in a western Guatemala population, raising suspicion that environmental factors like climactic factors, altitude, and UV exposure may contribute to XFS development and XFG progression [36].

These results do not completely explain the possibility of increased XFS for the Baja Verapaz, a mountainous region at 960 m and 15 N latitude with ample sunny days; thus, other factors may likely be at play. Further studies are warranted to understand possible environmental factors in various geographic locations that may be contributing to XFS and subsequent progression to XFG.

### 4.1. Other Considerations

A high prevalence of XFS/XFG of 36% was found in these patients seeking eye care at outreach clinics from 2016–2017. It is well accepted that XFS can lead to more advanced cataracts and vision threatening glaucoma and, thus, this cohort may have been more visually challenged and, therefore, seeking eye care. Family members with XFS/XFG create a substantial burden as the XFS/XFG family member likely cannot work and provide a necessary income to sustain members of the family if visually impacted. This may cause people outside and inside the home to be reliant on family members for resources. If there is a genetic or familial component, this could disproportionately affect families economically who currently have an XFS/XFG family member.

Barriers to accessible healthcare can be significant to this population and may include distance from medical providers, socioeconomic status, lack of transportation, and infrastructure. A single ophthalmologist could treat the 800,000 inhabitants near Salamá, but they are the inhabitants who can afford the care and who have the means and ability to travel. Some participants reported the difficulty to travel from their hometown to Salamá due to scarce daily bus trips. Taxis provide personalized timing and destination transportation, but are expensive compared to buses. Factoring in 1–2 days of travel for eye care means loss of income for those days, which is usually compounded by the need for a family member to accompany them. This is only a glimpse of the difficulties Guatemalans and other members of developing countries face in accessing healthcare.

Further, the patients that completed the trip to Salamá and nearby eye care communities camps were able to travel, which speaks to healthier more able-bodied individuals. This may not represent the population in greatest need of eye care due to poor health and those possibly lacking support rendering them unable to travel to Salamá or the nearby communities.

The Baja Verapaz region has a high concentration of indigenous Guatemalans that have historically been treated as lesser than their ladino (mixed Latino/European descent) counterparts [62]. This is an important consideration when analyzing the barriers to medical access in this region and how it may differ from that of other more urban or similarly rural regions of the country and South America. Bolivia has relatively similar dense indigenous populous regions where use of traditional medicine is employed due to limited access and the state of medical services for indigenous people fall short [63]. Another cultural consideration surrounding access to medical care in rural Guatemala is the history of the civil war. Guatemalans are still distrustful of authority and contemporary medical care, and this affects the decision to seek medical care—on top of transportation and financial concerns [62,64].

As part of the Moran Eye Center’s mission to improve access to ophthalmic eye care around the world, Dr. Orlando Gonzalez received hands-on training from Moran physicians and specialized training in utilizing phacoemulsification for cataract surgery.

### 4.2. Study Strengths

This is the largest study to date assessing the prevalence of XFS/XFG in the Guatemalan population of Baja Verapaz over the age of 15 yrs seeking eye exams and interventions.

Longitudinal follow up by Dr. Gonzalez continues to occur in managing ocular pathologies in the residents of Baja Verapaz. We hope to update this study in the future with ongoing results from Dr. Gonzalez.

### 4.3. Study Limitations

As this study was limited to self-selected patients who attended outreach eye camps seeking eye care, the sample size was modest. This study included community members from across the Baja Verapaz region, who were able to travel, that were found to have XFS, XFG, and/or other eye pathologies. Furthermore, given patients were being evaluated for surgery, and XFS is known to increase the likelihood of cataracts, we may be biasing the sample. Thus, our findings may not be representative of the general population of Baja Verapaz, but is likely representative of communities within and surrounding Salamá and in those with visual complaints as well as concomitant cataracts.

Another limitation is communication across an array of languages. The ability to obtain information depends on the quality of questions asked, the interpretation, and the understanding of the patients. No professional interpreters were available and clinic staff, family members, or outreach team members were available as interpreters for these encounters.

Further, this is a small population that was analyzed at a snapshot in time. Thus this study, with a relatively small sample size, may not have adequate power to detect small effects between groups, and we acknowledge that false-negative type II errors may have occurred. Future studies should have larger populations and longitudinally prospectively follow patients to better assess how various factors could affect XFS development and progression to XFG. Given the small sample size, such limitations exist due to population differences, study methodologies, lack of large population-based prospective studies, and differing environmental factors [65]. More data are needed to confirm what looks to be a high prevalence for XFS. It is important to acknowledge the significant impact on vision XFS can have, hence identifying this disease early could produce favorably impactful outcomes for a large portion of the population in the Baja Verapaz region.

## 5. Conclusions

This cross-sectional observational study demonstrates that the prevalence of XFS/XFG in Guatemalans in the Baja Verapaz region seeking eye care and interventions during a Moran Outreach trip was upwards of 36%. This rate is on the upper end compared to other populations globally and elevated compared to 15% in a western Guatemalan cataract population [36] and 24% in a Mayan cohort (22% also had cataracts) [38]. Larger epidemiology studies are warranted to better understand the prevalence and impact of XFS/XFG. Further, a next step could be to combine demographic and social factors from other papers as a meta-analysis to better understand risk for XFS and XFG.

## Figures and Tables

**Table 1:** Summary of demographics and comparison of XFS/XFG patients to non XFS/XFG patients.

		Diagnosis			
	Overall (N = 153)	XFS/XFG (N = 55)	Non XFS/XFG (N = 98)	*p*-Value	Bonferroni *p*-Value
Age in years, N (%)					
Median (IQR)	67.0 (57.0, 77.0)	74.0 (65.0, 79.0)	63.0 (47.0, 74.0)	<0.0001	0.0008
Mean (SD)	64.2 (16.8)	72.1 (9.5)	59.7 (18.4)		
(Min, Max)	(15, 94)	(48, 94)	(15, 87)		
Age group, N (%)					
<40	16 (10.5)	0 (0.0)	16 (16.3)	0.0002	
40 to 59	28 (18.3)	5 (9.1)	23 (23.5)		
60 to 69	38 (24.8)	13 (23.6)	25 (25.5)		
70 to 79	46 (30.1)	26 (47.3)	20 (20.4)		
≥80	25 (16.3)	11 (20.0)	14 (14.3)		
Gender, N (%)					
Male	66 (44.9)	28 (51.9)	38 (40.9)	0.1965	1.0000
Female	81 (55.1)	26 (48.1)	55 (59.1)		
Year, N (%)					
2016	92 (60.1)	36 (65.5)	56 (57.1)	0.3137	1.0000
2017	61 (39.9)	19 (34.5)	42 (42.9)		
Location, N (%)					
Rural	99 (64.7)	42 (76.4)	57 (58.2)	0.0238	0.5234
Urban	54 (35.3)	13 (23.6)	41 (41.8)		
Ethnicity, N (%)					
Mayan	57 (37.3)	22 (40.0)	35 (35.7)	0.7226	1.0000
Spanish	80 (52.3)	28 (50.9)	52 (53.1)		
Mayan & Spanish	14 (9.2)	5 (9.1)	9 (9.2)		
Don’t Know	2 (1.3)	0 (0.0)	2 (2.0)		
Occupation, N (%)					
Farmer	14 (23.0)	6 (31.6)	8 (19.0)	0.4670	1.0000
Housekeeper	33 (54.1)	10 (52.6)	23 (54.8)		
Other ^a^	14 (23.0)	3 (15.8)	11 (26.2)		
Outdoor occupation, N (%)					
No	37 (63.8)	11 (64.7)	26 (63.4)	0.9258	1.0000
Yes	21 (36.2)	6 (35.3)	15 (36.6)		
Parental Language, N (%)					
Spanish	110 (71.9)	35 (63.6)	75 (76.5)	0.0886	1.0000
Achí	44 (28.8)	22 (40.0)	22 (22.4)	0.0214	0.4700
Other ^b^	13 (8.5)	3 (5.5)	10 (10.2)	0.3120	1.0000
Grandparental Language, N (%)					
Spanish	93 (60.8)	31 (56.4)	62 (63.3)	0.4015	1.0000
Achí	47 (30.7)	22 (40.0)	25 (25.5)	0.0623	1.0000
Other ^b^	20 (13.1)	4 (7.3)	16 (16.3)	0.1109	1.0000
Past Medical History, N (%)					
Cardiovascular Disease and Hypertension	44 (28.8)	15 (27.3)	29 (29.6)	0.7610	1.0000
Diabetes	23 (15.0)	5 (9.1)	18 (18.4)	0.1234	1.0000
None	46 (30.1)	22 (40.0)	24 (24.5)	0.0447	0.9830
Other ^c^	81 (52.9)	30 (54.5)	51 (52.0)	0.7658	1.0000
Family History, N (%)					
Cardiovascular Disease and Hypertension	26 (17.0)	8 (14.5)	18 (18.4)	0.5459	1.0000
Asthma and Lung Disease	11 (7.2)	4 (7.3)	7 (7.1)	0.9762	1.0000
Diabetes	21 (13.7)	5 (9.1)	16 (16.3)	0.2120	1.0000
None	62 (40.5)	32 (58.2)	30 (30.6)	0.0009	0.0189
Other ^d^	58 (37.9)	16 (29.1)	42 (42.9)	0.0922	1.0000

Missing values: gender = 6, occupation = 92, and outdoor occupation = 95. ^a^ Other includes: Artisan, Athlete, Machine Worker, None, Police, Pool Worker, Seamstress, Security Guard, Student, Teacher, Welder. ^b^ Other includes: Ket(k)chí, Kaqchikel, Kikché, Mayan, Pokomchi, Don’t Know. ^c^ Other includes: ACL Surgery, Anemia, Appendectomy, Appendicitis, Arthralgia, Arthritis, Asthma, Back Pain, Bladder Disease, Bladder Stones, Blurry Vision, Bone Disorder, Breast Cancer, Breast Cyst, Bronchitis, C Section, Cataract, Cataract Right Eye, Cataracts, Cervical Adenoma, Chalazion Surgery, Cholecystectomy, Colds, Colitis, Colonic Cyst, Convulsions, Cough, Cramps, Dry Eyes, Eye Disorder, Femur/Knee Fracture, Fever, Flu, Gallbladder Disease, Gallbladder Pain, Gastritis, Glaucoma, Gout, Headaches, Hearing Loss, Hernia, Hypotension, Kidney Problems, Left Arm Tremor, Leg Fracture, Leg Pain, Liver Disease, Liver Problems, Lung Disorder, Malaria, Mastectomy, Myalgia In Extremities, Neck Surgery, OB/GYN Surgery, Ocular Surgery, Oophorectomy, Osteoporosis, Ovarian Cyst, Ovarian Disease, Ovarian Surgery, Pancreatitis, Paralysis, Prostate Disease, Prostate Surgery, Pterygium, Seasonal Allergies, Seizure, Stomach Ache, Stomach Cancer, Stomach Problems, Sun Allergy, Transient Ischemic Attack, Tubal Ligation, Tuberculosis, Uterine Disease, Uterine Surgery, Uterine Tumor, Uterus Problems, Vaginal Infection, Wrist Surgery. ^d^ Other includes: Alzheimer’s, Anemia, Appendectomy, Bilateral Cataracts, Bleeding Disorder, Blind, Bone Disorder, Breast Cancer, C Section, Cancer, Cataracts, Cholecystectomy, Chronic Cough, Cyst, Dystrophia, Esophageal Cancer, Eye Disorder, Facial Cyst, Fever, Gastric Cancer, Gastritis, Glaucoma, Headaches, Hearing Loss, Heart Attack, Leg Fracture, Liver Cancer, Myalgia In Extremities, Myopia, Osteoporosis, Pacemaker, Pelvis Fracture, Prostate Cancer, Prostate Disease, Pseudoexfoliation Syndrome, Recurrent Infections, Retinopathy, Seizure, Spine Fracture, Stomach Cancer, Stomach Pain, Stroke, Tonsillitis, Tracheal Cancer, Tuberculosis, Urinary Tract Infection, Weak Muscles.

**Table 2:** Multivariable regression model comparing demographics among XFS/XFG vs. non XFS/XFG patients.

	Univariate		Multivariate	
	Odds Ratios (95% CI)	*p*-Value	Odds Ratios (95% CI)	*p*-Value
Age in years	1.06 (1.03, 1.09)	<0.0001	1.06 (1.02, 1.09)	0.0003
Family History				
Yes	1.00		1.00	
No	3.15 (1.59, 6.27)	0.0010	2.34 (1.13, 4.83)	0.0221

## Data Availability

Data sharing is not applicable to this article.
